# Taxonomy of Phleboviruses, Emphasizing Those That Are Sandfly-Borne †

**DOI:** 10.3390/v13050918

**Published:** 2021-05-15

**Authors:** Charles H. Calisher, Mattia Calzolari

**Affiliations:** 1 Arthropod-Borne and Infectious Diseases Laboratory, Department of Microbiology, Immunology and Pathology, College of Veterinary Medicine and Biological Sciences, 3195 Rampart Rd., Foothills Campus, Colorado State University, Fort Collins, CO 80523-1690, USA; 2 Laboratorio Entomologia Sanitaria, Sede Territoriale di Reggio Emilia, Istituto Zooprofilattico Sperimentale della Lombardia e dell′Emilia Romagna “B. Ubertini” via Pitagora 2, 42124 Reggio Emilia, Italy; mattia.calzolari@izsler.it

**Keywords:** *Phlebovirus*, sand fly, reassortant virus, virus species

## Abstract

Sandfly-borne phleboviruses (phylum *Negarnavaricota*, realm *Riboviria*, kingdom *Orthornavirae*, genus *Phlebovirus*) comprise three genome segments of ribonucleic acid (RNA) and which encode an RNA-dependent RNA polymerase, which they use to transcribe the viral RNA genome into messenger RNA and to replicate the genome. At least some of these viruses cause mild 3-day fevers in humans but some also have been associated with more severe illnesses in humans. The 67 recognized phleboviruses are listed here in a table composed by the authors from International Committee on Taxonomy of Viruses reports as well as the scientific literature.

Viruses of the genus *Phlebovirus* (realm *Riboviria,* kingdom *Orthornaviridae*, phylum *Negarnavaricota*, order *Bunyavirales* and family *Phenuiviridae*) comprise three genome segments of ribonucleic acid (RNA) which encode an RNA-dependent RNA polymerase, which they use to transcribe the viral RNA genome into messenger RNA and to replicate the genome [1]. Phleboviruses have a large (L) segment (6.4 kb) that codes for the RNA-dependent RNA polymerase (RdRp); a medium (M) segment (3.2 kb), which encodes for several polyproteins, obtained by leaky scanning and cleaved into several proteins (Nsm-GN, Nsm, NSm′, Gn and Gc); and a small (S) segment (1.7 kb) that encodes for two proteins (N and NSs) with an ambisense strategy (https://viralzone.expasy.org/252 accessed on 10 April 2021).

The genus name is derived from Phlebotominae, the taxon of vectors of member species *sandfly fever* *Naples phlebovirus*, from the Greek phlebos, meaning “vein”. Species in the genus were previously defined by serological cross reactivity. The detection of new phleboviruses, not often available for serological assays, prompted the change of classification rules. Now, viral species are defined by 95% or greater identity in the amino acid sequences of their RdRp [TaxoProp 2019.026M.A.v1.Phenuiviridae_4gen79sp]. The genus currently comprises 67 species, listed in Table 1. Phleboviruses mentioned in this Special Issue have not all been detected in sandflies. Those that have are noted in Table 1 with an asterisk. Some of these viruses have other hematophagous arthropods as their main vectors, such as mosquitoes for Rift Valley fever virus, while Mukawa virus has been isolated from ticks but remains in the genus *Phlebovirus*, despite the observations that most tick-borne viruses formerly included in the genus *Phlebovirus* are now included in the genus *Uukuvirus*. Some phleboviruses have been isolated from vertebrates, such as wild or sentinel rodents in the Americas, and in Africa, such as opossums or sloths. Other phleboviruses have been isolated from febrile patients in South America (Table 1). This variety of sources highlights the possible presence of diverse epidemiological cycles of these viruses. A high rate of vertical transmission of Toscana virus has been demonstrated in sandflies by experimental infections [2,3], suggesting that there is an amplifying role for vertebrate hosts but that maintenance in nature is mainly by sandflies. 

“Sandfly” (or “sand fly”) is a colloquial name for members of any species or genus of flying, biting, blood-sucking dipteran encountered in sandy areas. In the United States, “sandfly” may refer to certain horse flies that are also known as “greenheads”, or to members of the family Ceratopogonidae. Outside the United States, “sandfly” may refer to members of the subfamily Phlebotominae within the Psychodidae. The three main genera are *Lutzomyia* (found in the New World) and *Phlebotomus* and *Sergentomyia* (both found in the Old World), the former two genera contain the more relevant species able to transmit viral pathogens [4]. Biting midges are sometimes called “sandflies” or “no-see-ums”. New Zealand sandflies are in the genus *Austrosimulium*, a type of black fly (https://en.wikipedia.org/wiki/Sandfly accessed on 10 April 2021).

Infections with many of these viruses cause mild 3-day fevers, also known as pappataci fevers or phlebotomus fevers [5]. These illnesses are influenza-like and are characterized by a rapid onset. The diseases occur commonly in endemic areas in summer months, especially in August during which sandflies are active. Toscana virus has been associated with benign meningitis and, occasionally, more severe meningitis in humans [6]. The most important phlebovirus is Rift Valley fever virus, which has been responsible for wide-spread epidemics and epizootics in livestock in Africa, most notably in Egypt [7]. However, it is transmitted principally by mosquitoes, and so it is not mentioned further here.

A diagnostically complicating feature of phlebovirus replication may be reassortant generation resulting from multiple simultaneous phlebovirus infections [8]. As with other segmented RNA viruses, the reassortment of RNA segments of phleboviruses is commonly observed. By this means, the RNA segments of different virus strains become mixed during replication, and the progeny viruses contain genome segments of the parental viruses. Thus, the progeny viruses have new combinations of these segments and possess novel properties and may be confused for one another due to the specificity of the testing procedures. Only complete genetic analyses can be used to definitively identify such progeny [9]. It has been argued that perhaps all available viruses in this virus family may be the most recent of long genetic lineages [8]. Undoubtedly, some (or all) of the viruses listed in Table 1 are reassortant phleboviruses, particularly those detected in Italy [10], possibly due to the co-circulation of multiple phleboviruses in arthropod vectors occurring in close proximity [11].

To assess possible reassortant phleboviruses, amino acid sequences of the RdRp and correspondent M segments have been retrieved from GenBank and aligned with MAFFT [12]. The percentage of identity has been evaluated with MegaX software, using p-distance with a pairwise deletion option [13]. A maximum likelihood phylogenetic tree was obtained with the RdRp aligned using IQtree software [14]. In this tree, in Figure 1, sequences with more than 95% identity, then ascribable to a single species, are highlighted in red.

The likely reassortant phleboviruses have different M segments (with the exception of Ponticelli II and Bregalaka, which are very similar). Reassortment events have been described for phleboviruses of the Candiru antigenic complex [15], among Massilia, Granada and Arrabida viruses [16,17], and likely produced Ponticelli I, Ponticelli II and Ponticelli III, which belong to the *Adana phlebovirus* species, according to the RdRp threshold. The possibility of reassortment involving the M segment would be a relevant phenomenon in the evolution of this group, as similarly reported for orthobunyaviruses [18]. The M segment is likely responsible for modifying the pathogenic potential of a virus, as has been reported for reassortant orthobunyaviruses [18,19].

**Table 1:** Viruses of the genus *Phlebovirus,* modified from [1].

Species ^1^	Virus ^2^	Abbreviation
*Adana phlebovirus*	Adana virus *	ADAV
	Ponticelli I virus *	
	Ponticelli II virus *	
	Ponticelli III virus *	
	Bregalaka virus *	
*Aguacate phlebovirus*	Aguacate virus *	AGUV
*Alcube phlebovirus*	Alcube virus *	ACBV
*Alenquer phlebovirus*	Alenquer virus	ALEV
*Ambe phlebovirus*	Ambe virus *	ABEV
*Anhanga phlebovirus*	Anhangá virus	ANHV
*Arumowot phlebovirus*	Arumowot virus	AMTV
*Bogoria phlebovirus*	Bogoria virus	BGRV
*Buenaventura phlebovirus*	Buenaventura virus *	BUEV
*Bujaru phlebovirus*	Bujaru virus	BUJV
*Cacao phlebovirus*	Cacao virus *	CACV
*Campana phlebovirus*	Campana virus *	CMAV
*Candiru phlebovirus* ^3^		
	Ariquemes virus	ARQV
	Candirú virus	CDUV
	Jacundá virus	JCNV
	Morumbi virus	MRBV
	Mucura virus	MCRV
	Serra Norte virus	SRNV
*Chagres phlebovirus*	Chagres virus *	CHGV
*Cocle phlebovirus*	Coclé virus	CCLV
*Corfou phlebovirus*	Corfou virus *	CFUV
*Dashli phlebovirus*	Dāshlī virus *	DASV
*Durania phlebovirus*	Durania virus *	DRNV
*Echarate phlebovirus*	Echarate virus	ECHV
*Embossos phlebovirus*	Embossos virus *	EMBV
*Gabek phlebovirus*	Gabek forest virus	GFV
*Gordil phlebovirus*	Gordil virus	GORV
*Icoaraci phlebovirus*	Icoaraci virus	ICOV
*Itaituba phlebovirus*	Itaituba virus	ITAV
*Itaporanga phlebovirus*	Itaporanga virus	ITPV
*Ixcanal phlebovirus*	Ixcanal virus *	IXCV
*Karimabad phlebovirus*	Karimabad virus *	KARV
*Kiborgoch phlebovirus*	Kiborgoch virus *	KBGV
*La Gloria phlebovirus*	La Gloria virus *	LAGV
*Lara phlebovirus*	Rio Claro virus	RICV
*Leticia phlebovirus*	Leticia virus *	LTCV
*Maldonado phlebovirus*	Maldonado virus	MLOV
*Mariquita phlebovirus*	Mariquita virus *	MRQV
*Massilia phlebovirus*	Massilia virus *	MASV
*Medjerda phlebovirus*	Medjerda Valley virus *	MVV
*Mona Grita phlebovirus*	Mona Grita virus *	MOGV
*Mukawa phlebovirus*	Mukawa virus	MKWV
*Munguba phlebovirus*	Munguba virus *	MUNV
*Naples phlebovirus* ^3^		
	Arrabida virus *	ARRV
	Balkan virus *	BALKV
	Fermo virus *	FERV
	Granada virus *	GRAV
	Saddaguia virus *	SADV
	sandfy fever Naples virus *	SFNV
*Nique phlebovirus*	Nique virus *	NIQV
*Ntepes phlebovirus*	Ntepes virus *	NTPV
*Odrenisrou phlebovirus*	Odrénisrou virus	ODRV
*Oriximina phlebovirus*	Oriximiná virus *	ORXV
*Pena Blanca phlebovirus*	Peña Blanca virus *	PEBV
*Penshurt phlebovirus*	Penshurt virus	PEHV
*Perkerra phlebovirus*	Perkerra virus	PKEV
*Punique phlebovirus*	Punique virus*	PUNV
*Punta Toro phlebovirus* ^3^		
	Buenaventura virus *	BUEV
	Capira virus *	CAPIV
	Punta Toro virus *	PTV
*Rift Valley fever phlebovirus* ^4^	Rift Valley fever virus	RVFV
	*Hedi virus* * [20]	HEDV
*Rio Grande phlebovirus*	Rio Grande virus	RGV
*Saint Floris phlebovirus*	Saint-Floris virus	SAFV
*Salanga phlebovirus*	Salanga virus	SLGV
*Salehabad phlebovirus* ^3^		
	Adria virus *	ADRV
	Arbia virus *	ARBV
	Olbia virus *	OLBV
	Salehabad virus *	SALV
	Zaba virus *	ZABAV
*Salobo phlebovirus*	Salobo virus *	SLBOV
*Sicilian phlebovirus*	sandfy fever Sicilian virus *	SFSV
*Tapara phlebovirus*	Tapará virus *	TPRV
*Tehran phlebovirus*	Tehran virus *	THEV
*Tico phlebovirus*	Tico virus *	TICV
*Toros phlebovirus*	Toros virus *	TORV
*Toscana phlebovirus*	Toscana virus *	TOSV
*Tres Almendras phlebovirus*	Tres Almendras virus *	TRAV
*Turuna phlebovirus*	Turuna virus *	TUAV
*Uriurana phlebovirus*	Uriurana virus *	URIV
*Urucuri phlebovirus*	Urucuri virus	URUV
*Viola phlebovirus*	Viola virus *	VIOV
*Zerdali phlebovirus*	Zerdali virus *	ZERV

^1^ Taxon names are always italicized and always begin with a capital letter. Note that viruses are real objects that are assigned to concepts that are called taxa. Species, genera, subfamilies, families and orders are taxa; ^2^ virus names are not italicized and are not capitalized, except if the name or a name component is a proper noun. This column lists the virus names with their correct (including lack of) capitalization; ^3^ lists of viruses within a given species are provisional at this point and will likely be amended in the near future; ^4^ type of species; * detected in sandflies.

## Figures and Tables

**Figure 1:**
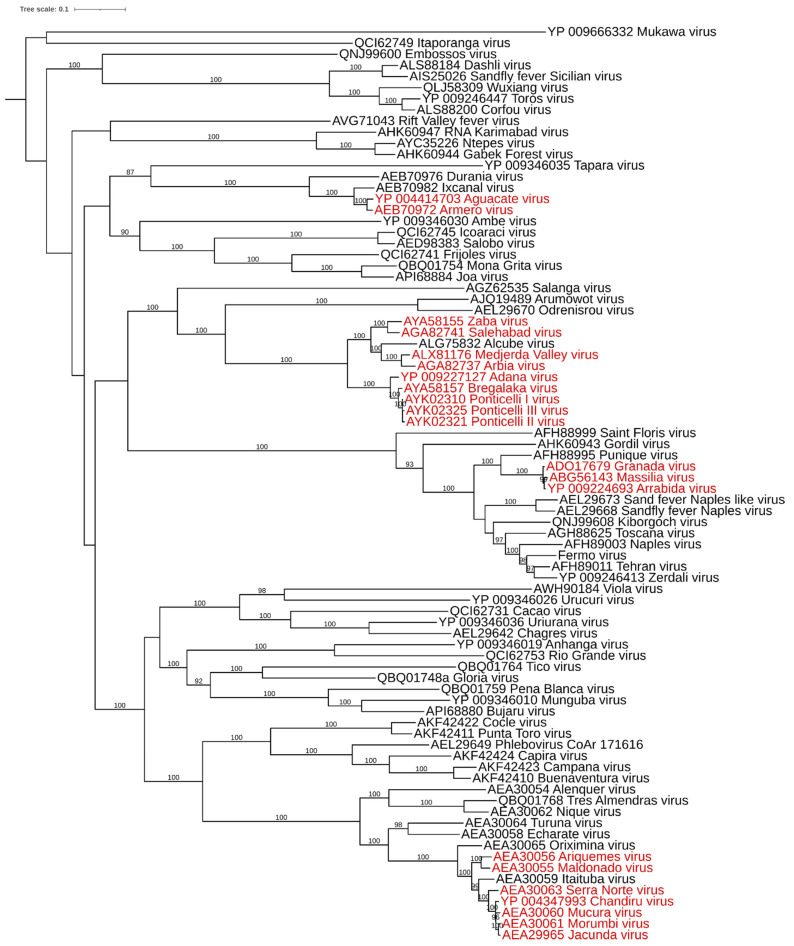
Midpoint rooted maximum likelihood tree obtained with amino acid sequences of the RdRp of available phleboviruses. Sequences in red: ascribable to the same species according the 95% identity threshold. Bootstrap values >85% are shown over the branch.

## References

[1] Kuhn J.H., Adkins S., Alioto D., Alkhovsky S.V., Amarasingh G.K., Anthony S.J., Avšič-Županc T., Ayllón M.A., Bahl J., Balkema-Buschmann A. (2020). 2020 taxonomic update for phylum *Negarnaviricota* (*Riboviria*: *Orthornavirae*), including the large orders *Bunyavirales* and *Mononegavirales*. Arch. Virol..

[2] Tesh R.B., Modi G.B. (1987). Maintenance of Toscana virus in *Phlebotomus perniciosus* by vertical transmission. Am. J. Trop. Med. Hyg..

[3] Maroli M., Ciufolini M.G., Verani P. (1993). Vertical transmission of Toscana virus in the sandfly, *Phlebotomus perniciosus*, via the second gonotrophic cycle. Med. Vet. Entomol..

[4] Service M.W. (2001). Phlebotomine sand-flies (Phlebotominae). The Encyclopedia of Arthropod-Transmitted Infections.

[5] Ashford R.W., Service M.W. (2001). Phlebotomus fevers. The Encyclopedia of Arthropod-Transmitted Infections.

[6] Calisher C.H., Weinberg A.N., Muth D.J., Lazuick J.S. (1987). Toscana virus infection in United States citizen returning from Italy. Lancet.

[7] Young P.R., Ng L.F.P., Hall R.A., Smith D.W., Johansen C.A., Farrar J. (2013). Arbovirus Infections. Manson’s Tropical Diseases: Twenty-Third Edition.

[8] Briese T., Calisher C.H., Higgs S. (2013). Viruses of the family *Bunyaviridae*: Are all available isolates reassortants?. Virology.

[9] Borucki M.K., Chandler L.J., Parker B.M., Blair C.D., Beaty B.J. (1999). Bunyavirus superinfection and segment reassortment in transovarially infected mosquitoes. J. Gen. Virol..

[10] Calzolari M., Chiapponi C., Bellini R., Bonilauri P., Lelli D., Moreno A., Barbieri I., Pongolini S., Lavazza A., Dottori M. (2018). Isolation of three novel reassortant phleboviruses, Ponticelli I, II, III, and of Toscana virus from field-collected sand flies in Italy. Parasites Vectors.

[11] Calzolari M., Ferrarini G., Bonilauri P., Lelli D., Chiapponi C., Bellini R., Dottori M. (2018). Co-circulation of eight different phleboviruses in sand flies collected in the Northern Apennine Mountains (Italy). Infect. Genet. Evol..

[12] Katoh K., Rozewicki J., Yamada K.D. (2019). MAFFT online service: Multiple sequence alignment, interactive sequence choice and visualization. Brief. Bioinform..

[13] Kumar S., Stecher G., Li M., Knyaz C., Tamura K. (2018). MEGA X: Molecular Evolutionary Genetics Analysis across computing platforms. Mol. Biol. Evol..

[14] Trifinopoulos J., Nguyen L.T., von Haeseler A., Minh B.Q. (2016). W-IQ-TREE: A fast online phylogenetic tool for maximum likelihood analysis. Nucleic Acids Res..

[15] Palacios G., Tesh R., Travassos da Rosa A., Savji N., Sze W., Jain K., Serge R., Guzman H., Guevara C., Nunes M.R. (2011). Characterization of the Candiru antigenic complex (*Bunyaviridae*: *Phlebovirus*), a highly diverse and reassorting group of viruses affecting humans in tropical America. J. Virol..

[16] Collao X., Palacios G., de Ory F., Sanbonmatsu S., Pérez-Ruiz M., Navarro J.M., Molina R., Hutchison S.K., Lipkin W.I., Tenorio A. (2010). Granada virus: A natural phlebovirus reassortant of the sandfly fever Naples serocomplex with low seroprevalence in humans. Am. J. Trop. Med. Hyg..

[17] Amaro F., Hanke D., Zé-Zé L., Alves M.J., Becker S.C., Höper D. (2016). Genetic characterization of Arrabida virus, a novel phlebovirus isolated in South Portugal. Virus Res..

[18] Briese T., Bird B., Kapoor V., Nichol S.T., Lipkin W.I. (2006). Batai and Ngari viruses: M segment reassortment and association with severe febrile disease outbreaks in East Africa. J. Virol..

[19] Gerrard S.R., Li L., Barrett A.D., Nichol S.T. (2004). Ngari virus is a Bunyamwera virus reassortant that can be associated with large outbreaks of hemorrhagic fever in Africa. J. Virol..

[20] Ziqian X., Fan N., Hou X., Wang J., Fu S., Song J., Shi M., Liang G. (2021). Isolation and identification of a novel phlebovirus, Hedi virus, from sandflies collected in China. Viruses.

